# Antigenicity and immunogenicity of PvRALP1, a novel *Plasmodium vivax* rhoptry neck protein

**DOI:** 10.1186/s12936-015-0698-z

**Published:** 2015-04-29

**Authors:** Yang Cheng, Jian Li, Daisuke Ito, Deok-Hoon Kong, Kwon-Soo Ha, Feng Lu, Bo Wang, Jetsumon Sattabongkot, Chae Seung Lim, Takafumi Tsuboi, Eun-Taek Han

**Affiliations:** Department of Medical Environmental Biology and Tropical Medicine, Kangwon National University School of Medicine, Hyoja2-dong, Chuncheon, Gangwon-do 200-701 Republic of Korea; Laboratory of Malaria and Vector Research, National Institute of Allergy and Infectious Diseases (NIAID), National Institutes of Health (NIH), Rockville, MD 20852 USA; Department of Parasitology, College of Basic Medicine, Hubei University of Medicine, Hubei, 442000 China; Division of Malaria Research, Proteo-Science Center, Ehime University, Matsuyama, Ehime 790-8577 Japan; Department of Molecular and Cellular Biochemistry, School of Medicine, Kangwon National University, Chuncheon, Gangwon-do 200-701 Republic of Korea; Key Laboratory of Parasitic Disease Control and Prevention (Ministry of Health), and Jiangsu Provincial Key Laboratory of Parasite Molecular Biology, Jiangsu Institute of Parasitic Diseases, Wuxi, Jiangsu Province People’s Republic of China; Department of Clinical Laboratory, The First Affiliated Hospital of Anhui Medical University, Hefei, Anhui People’s Republic of China; Mahidol Vivax Research Unit, Faculty of Tropical Medicine, Mahidol University, Bangkok, 10400 Thailand; Department of Laboratory Medicine, College of Medicine, Korea University Guro Hospital, Seoul, 152-703 Republic of Korea

**Keywords:** *Plasmodium vivax*, RALP1, Rhoptry neck protein, Antigenicity, Immunogenicity

## Abstract

**Background:**

Proteins secreted from the rhoptry in *Plasmodium* merozoites are associated with the formation of tight junctions and parasitophorous vacuoles during invasion of erythrocytes and are sorted within the rhoptry neck or bulb. Very little information has been obtained to date about *Plasmodium vivax* rhoptry-associated leucine (Leu) zipper-like protein 1 (PvRALP1; PVX_096245), a putative rhoptry protein. PvRALP1 contains a signal peptide, a glycine (Gly)/glutamate (Glu)-rich domain, and a Leu-rich domain, all of which are conserved in other *Plasmodium* species.

**Methods:**

Recombinant PvRALP1s were expressed as full-length protein without the signal peptide (PvRALP1-Ecto) and as truncated protein consisting of the Gly/Glu- and Leu-rich domains (PvRALP1-Tr) using the wheat germ cell-free expression system. The immunoreactivity to these two fragments of recombinant PvRALP1 protein in serum samples from *P. vivax*-infected patients and immunized mice, including analysis of immunoglobulin G (IgG) subclasses, was evaluated by enzyme-linked immunosorbent assay or protein microarray technology. The subcellular localization of PvRALP1 in blood stage parasites was also determined.

**Results:**

Recombinant PvRALP1-Ecto and PvRALP1-Tr proteins were successfully expressed, and in serum samples from *P. vivax* patients from the Republic of Korea, the observed immunoreactivities to these proteins had 58.9% and 55.4% sensitivity and 95.0% and 92.5% specificity, respectively. The response to PvRALP1 in humans was predominantly cytophilic antibodies (IgG1 and IgG3), but a balanced Th1/Th2 response was observed in mice. Unexpectedly, there was no significant inverse correlation between levels of parasitaemia and levels of antibody against either PvRALP1-Ecto (*R*^2^ = 0.11) or PvRALP1-Tr (*R*^2^ = 0.14) antigens. PvRALP1 was localized in the rhoptry neck of merozoites, and this was the first demonstration of the localization of this protein in *P. vivax*.

**Conclusions:**

This study analysed the antigenicity and immunogenicity of PvRALP1 and suggested that PvRALP1 may be immunogenic in humans during parasite infection and might play an important role during invasion of *P. vivax* parasites.

## Background

Malaria is a disease caused by plasmodial parasite infection of host erythrocytes. The blood stage of *Plasmodium* causes the pathobiology of malaria by invasion and subsequent modification of human erythrocytes. Therefore, the search for candidate vaccine antigens against malaria parasites has mainly focused on blood-stage parasite antigens, especially those located on the surface or in the apical organelles of the merozoite, such as rhoptries and micronemes [1]. In the case of *Plasmodium vivax*, using serological assays, a number of blood-stage proteins that are potential blood-stage vaccine candidates were screened in our previous study [2]. The antigenicity, immunogenicity, function, and subcellular localization of these proteins including merozoite surface protein 1 paralog (MSP1P) [3,4], Pv41 [5], PvMSP10 [6], Pv12 [7], and RhopH2 [8] were also evaluated.

Apicomplexan parasites, such as *Toxoplasma gondii* and *Plasmodium* species, actively invade host cells through a moving junction (MJ) complex assembled at the parasite–host cell interface [9]. Major apical organelle proteins are involved in this serial invasion process, and the rhoptry neck protein RON complex together with the micronemal protein AMA1 forms the MJ [10,11]. However, some rhoptry proteins are released during invasion and migrate to the lumen or membrane of the nascent parasitophorous vacuole or the interior of the host cell, rather than to the MJ [12].

The rhoptry-associated leucine (Leu) zipper-like protein 1 of *Plasmodium falciparum* (PfRALP1) was first identified by a high degree of protein sequence homology among field isolates, and translocates from the rhoptry neck to the MJ during merozoite invasion [13]. Attempts to knock out *pfralp1* were unsuccessful [14], which suggests that it might play an important role in invasion of malaria parasites. Recently, an erythrocyte-binding epitope in the C-terminal region of PfRALP1 was identified, it was shown that anti-RALP1 antibodies disrupted MJ formation, and growth and invasion inhibition assays confirmed the important role of PfRALP1 during merozoite invasion of erythrocytes [13]. Six orthologs of PfRALP1 have been found in different *Plasmodium* species [15]. Comparative analysis of the deduced amino acid sequences of the PfRALP1 and *P. vivax* RALP1 (PvRALP1) revealed an overall sequence identity of ~67% and similarity of ~83% [16]. Through liquid chromatography-tandem mass spectrometry, PvRALP1 has been identified in clinical isolates [17,18] and the VCG-1 strain, and *in silico* modelling predicted it as a vaccine candidate [19]. All RALP1 orthologs include coiled-coil region(s); these regions are targets for antibody recognition and these antibodies may be possibly protective [20].

Profiling of PfRALP1 has shown its robust immunogenicity among blood-stage antigens of *P. falciparum* [13,21]. As an ortholog of PfRALP1, PvRALP1 is also likely to be immunogenic during malaria parasite infection in humans [16]. In this study, strong antigenicity and immunogenicity of PvRALP1, and its localization in the rhoptry neck of merozoites of *P. vivax* were demonstrated.

## Methods

### Blood samples of *Plasmodium vivax* patients

A total of 112 blood samples (mean parasitaemia 0.117%, range 0.002–0.630%) were obtained from patients who were confirmed positive for *P. vivax* malaria via microscopy at Kangwon National University Hospital and at local health centres and clinics in Gangwon Province, which is a malaria-endemic area of the Republic of Korea. The mean age of patients was 27 years (range 18–61 years). Eighty blood samples were also taken from healthy individuals in nonmalaria-endemic areas, who were confirmed negative for *P. vivax* malaria by microscopy, and had no previous history of malaria. This study was approved by the Institutional Review Board at Kangwon National University Hospital and all the blood samples were collected after obtaining informed consent.

### Enrichment of parasite-infected erythrocytes for parasite antigen

*Plasmodium vivax*-infected blood samples were collected from patients and parasite-infected erythrocytes were purified as reported previously [22]. White blood cells were removed from infected patient samples using a Plasmodipur filter (Euro-Diagnostica, Arnhem, The Netherlands), and the erythrocytes resuspended in RPMI-1640 medium (Invitrogen, Carlsbad, CA, USA) to make a 10% haematocrit suspension. Thereafter, schizont-rich infected erythrocytes were enriched by 60% Percoll gradient centrifugation and used as a source of parasite antigens for western blot and immunofluorescence analyses.

### Expression and purification of recombinant PvRALP1 proteins

Genomic DNA was prepared from Korean isolates of *P. vivax* as described previously [16]. The full-length of *pvralp1* comprising amino acid 1 to 675 was amplified from genomic DNA with the forward primer RALP1-F: 5′-ATGAAGCGGAGCATCGC-3′ and reverse primer RALP1-R: 5′-CTAGAACATGTCGTAGAGCAGGC-3′. The PCR amplification was performed on a MyCycler Thermal Cycler (Bio-Rad, Hercules, CA, USA) using the following temperature profile: 95°C for 4 min; 30 cycles at 95°C for 30 sec, 53°C for 30 sec, 72°C for 2 min; and a final extension at 72°C for 10 min. The resulting PCR product was cloned into the pCR2.1 TOPO vector (Invitrogen). Automated DNA sequence analysis of the cloned vector was performed using an ABI Prism 3730XL DNA Sequencer (Applied Biosystems, Foster City, CA, USA). The predicted protein domains of PvRALP1 were further analysed using the Simple Modular Architecture Research Tool (SMART) [23] and SOSUIsignal [24].

To express the two recombinant PvRALP1 proteins, the open reading frame of *pvralp1* without the signal peptide sequence (*pvralp1-ecto*; comprising amino acids 31 to 675) was amplified with the forward primer RALP1-Ecto F: 5′-atcactagtt*ctcgag*ATGGCGTACCGCCTAAAGAGG-3′ and the reverse primer RALP1-Ecto R: 5′-ccctatatat*ggatcc*TCACTAGAACATGTCGTAGAGCAGGC-3′, and the truncated *pvralp1* (*pvralp1-tr*; comprising amino acids 257 to 503) with a hexa-histidine (His)-tag at the C-terminus was amplified with the forward primer RALP1-Tr-F: 5′-atcactagtt*ctcgag*ATGACCTACGCGAGCTACGAAC-3′ and reverse primer RALP1-Tr-R: 5′-ccctatatat*ggatcc*TCAGTGATGATGATGATGATGTCAATTTAGCAAATTAGAGACGATGTTCTG-3′. The vector sequences are shown in lowercase, and the restriction enzyme sites (*Xho*I for sense primers and *Bam*HI for antisense primers) in italics. The underlined sequences in the antisense primers above indicate the regions that encode the His-tag. The amplified DNA sequences of *pvralp1-ecto* and *pvralp1-tr* were cloned into the *Xho*I and *Bam*HI sites of the pEU-E01-GST-TEV-MCS-N2 and pEU-E01-MCS vectors (CellFree Sciences, Matsuyama, Japan), respectively. Plasmid DNA was then prepared using the Maxi Plus™ Ultrapure plasmid extraction system (Viogene, Taipei, Taiwan) according to the manufacturer’s instructions. Purified plasmid DNA was eluted in 0.1× TE buffer (10 mM Tris–HCl, pH 8.0, 1 mM EDTA) and used for *in vitro* transcription for recombinant protein expression in the wheat germ cell-free (WGCF) system (CellFree Sciences). Glutathione *S*-transferase (GST) fusion PvRALP1-Ecto protein was purified with a glutathione-Sepharose 4B column according to the manufacturer’s instructions (GE Healthcare, Camarillo, CA, USA). PvRALP1-Tr protein with a His-tag at the C-terminus was purified using nickel nitrile-triacetic acid (Ni-NTA) affinity chromatography as described elsewhere [25].

### Immunization of mice and rabbit with recombinant PvRALP1s

To generate antibodies against PvRALP1-Ecto or PvRALP-Tr, three BALB/c mice for each protein were immunized subcutaneously with 20 μg per mouse of purified protein in Freund’s complete adjuvant, followed by two injections of 20 μg in Freund’s incomplete adjuvant three weeks and six weeks later. In addition, one Japanese white rabbit was immunized subcutaneously with 250 μg of purified PvRALP1-Tr in Freund’s complete adjuvant, followed by two injections of 250 μg in Freund’s incomplete adjuvant three weeks and six weeks later. The antisera were collected 14 days after the last immunization. Animal experimental protocols were approved by the Institutional Animal Care and Use Committee of Ehime University and Kangwon National University, and the experiments were conducted according to the Ethical Guidelines for Animal Experiments of Ehime University and Kangwon National University.

### SDS-PAGE and western blot analysis

The parasite proteins were extracted in reducing sample buffer for SDS-PAGE. Five micrograms of recombinant PvRALP1-Ecto or PvRALP1-Tr protein were loaded into each well and separated by SDS-PAGE under reducing conditions. The separated proteins were transferred to 0.45 μm polyvinylidene fluoride membranes (Millipore, Billerica, MA, USA) in a semidry transfer buffer (50 mM Tris, 190 mM glycine, 3.5 mM SDS, 20% methanol) at 400 mA for 40 min using a semidry blotting system (ATTO Corp., Tokyo, Japan). After blocking with 5% skim milk in phosphate-buffered saline containing 0.2% Tween 20 (PBS-T), the membranes were probed with mouse anti-PvRALP1-Ecto and anti-PvRALP1-Tr sera, rabbit anti-PvRALP1-Tr serum, anti-GST monoclonal antibody (Novagen, Madison, WI, USA), anti-penta-His monoclonal antibody (Qiagen), preimmune mouse serum, pooled sera from *P. vivax* malaria patients or noninfected individuals, all diluted 1:200 in PBS-T. IRDye goat anti-mouse, IRDye goat anti-rabbit, or IRDye goat anti-human sera (LI-COR Biosciences, Lincoln, NE, USA) were used to detect recombinant proteins according to the manufacturer’s instructions. Data were scanned with an Odyssey infrared imaging system (LI-COR Biosciences) and analysed with Odyssey software (LI-COR Biosciences).

### Serum screening using protein arrays

Sera from 112 patients with *P. vivax* malaria and 80 healthy individuals were tested against the recombinant PvRALP1-Ecto and PvRALP1-Tr proteins using protein arrays. A series of doubling dilutions was used to optimize the coating antigen concentration (3 to 200 ng/μL) of each recombinant protein. As a result, one microlitre of 50 ng/μL recombinant PvRALP1-Ecto or PvRALP1-Tr proteins were spotted onto each well of an amino-functionalized slide and incubated for 2 h at 37°C. The arrays were first blocked with 5% bovine serum albumin in PBS-T for 1 h at 37°C, then they were probed with human serum (1:10) that was preabsorbed against wheat germ lysate (1:100) to remove anti-wheat germ antibodies. The arrays were incubated with serum in PBS-T for 1 h at 37°C and antibodies were visualized with 10 ng/μL Alexa Fluor 546 goat anti-human immunoglobulin G (IgG; Invitrogen) diluted in PBS-T, and scanned in a fluorescence scanner (ScanArray Express; PerkinElmer, Boston, MA, USA) [7]. Fluorescence intensities of array spots were quantified by the fixed-circle method using ScanArray Express software (version 4.0; PerkinElmer). The positive cutoff value was calculated as the mean fluorescence intensity (MFI) of the negative controls plus 2 standard deviations (SD).

To investigate the human IgG subclasses comprising the response against PvRALP1-Ecto and PvRALP1-Tr, sera from fifty *P. vivax*-positive patients from malaria-endemic areas and ten *P. vivax*-negative sera from malaria nonendemic areas of Korea were randomly selected. One microlitre of 50 ng/mL PvRALP1-Ecto and PvRALP1-Tr proteins were spotted onto each well and incubated for 2 h at 37°C. After blocking, plasma was added in duplicate at previously determined dilutions. For measurement of IgG subclasses, mouse monoclonal antibodies to human IgG subclasses (IgG1, clone HP6096, IgG2 clone HP6002, IgG3 clone HP6047, and IgG4 clone HP6025 [Invitrogen]) as secondary antibodies were added at a dilution of 1:1,000. Alexa Fluor 546 goat anti-mouse antibody (Invitrogen) was added at 50 ng/μL as the tertiary antibody for the subclass assays. Finally, the data from scanned images were analysed as above.

### Enzyme-linked immunosorbent assay (ELISA)

To compare the IgG subclasses in anti-PvRALP1 and anti-PvRALP1-Tr immune mouse sera, PvRALP1-Ecto (5 μg/mL) or PvRALP1-Tr (5 μg/mL) was coated on 96-well ELISA plates. One hundred microlitres of purified mouse IgG1, IgG2a, IgG2b, and IgG3 (BD Pharmingen Corp., San Diego, CA, USA) as standards were also coated on 96-well plates at 256, 128, 64, 32, 16, 8, and 4 ng/mL. The coated plates were incubated with immune mouse sera diluted 1:1,000 in PBS-T. After washing, the plates were incubated with horseradish peroxidase-conjugated anti-mouse IgG1, IgG2a, IgG2b, and IgG3 antibodies (Invitrogen) at 1:1,000, 1:1,000, 1:2,000, and 1:1,000 dilutions, respectively. After washing, the plates were incubated with 100 μL of tetramethylbenzidine solution and the absorbance at 450 nm was measured within 1 h after addition of the stop solution. The concentration of each IgG subclass was calculated using a log − log curve fit.

### Indirect immunofluorescence assay (IFA)

IFAs were performed on acetone-fixed parasites as described previously [7]. The following primary antibody dilutions were used: mouse anti-PvRALP1-Tr (1:50), rabbit anti-PvRON2 (1:100), rabbit anti-PvDBP (1:100), and rabbit anti-PvRhopH2 (1:100). The following secondary antibodies were used: Alexa Fluor 488 goat anti-mouse IgG (1:500; Invitrogen), Alexa Fluor 546 goat anti-rabbit IgG (1:500; Invitrogen) and 4′,6′-diamidino-2-phenylindole (DAPI) for nuclear staining (1:1,000; Invitrogen). The slides were mounted in ProLong Gold antifade reagent (Invitrogen) and visualized under oil immersion in a confocal scanning laser microscope (LSM710; Carl Zeiss MicroImaging, Thornwood, NY, USA) using a Plan-Apochromat 63×/1.4 oil differential interference contrast (DIC) objective lens. Images were captured with Zen software (Carl Zeiss MicroImaging) and processed with Adobe Photoshop (Adobe Systems, San Jose, CA, USA).

### Statistical analyses

Simple scatter regression was used to construct a standard curve using SigmaPlot (Systat Software Inc., San Jose, CA, USA). Data were analysed using GraphPad Prism (GraphPad Software, San Diego, CA, USA); Student’s *t*-test or one-way ANOVA was used to evaluate the differences between the means of each group. Differences with *p* < 0.05 were considered significant.

## Results

### *Pvralp1* gene structure

**Figure 1:**
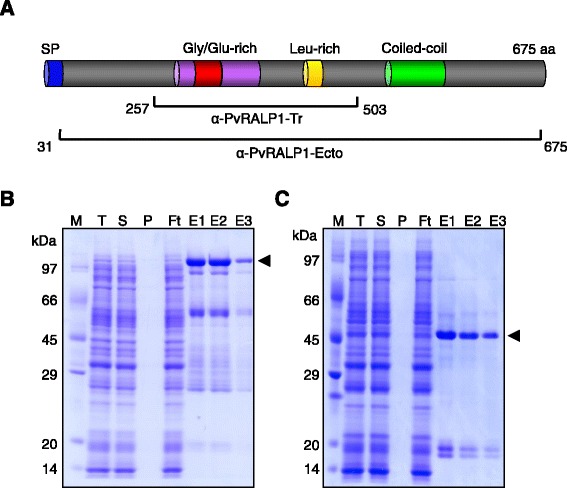
Schematic diagram and protein expression of PvRALP1-Ecto and PvRALP1-Tr. **(A)** Schematic diagram of PvRALP1-Ecto and the central domain comprising PvRALP1-Tr. **(B)** Expression and purification of recombinant PvRALP1-Ecto and **(C)** PvRALP1-Tr fragments. Arrowheads indicate specific bands for each recombinant protein. M, protein marker; T, total translation mix; S: supernatant; P, pellet; Ft: flow through; E1, elution fraction 1; E2, elution fraction 2; E3, elution fraction 3.

The gene sequence and transcription profiles of *pvralp1* (PlasmoDB accession no. PVX_096245) have been deposited at the PlasmoDB Web site [26]. Because of the lack of information about whether PvRALP1 contained a signal peptide*,* the *pfralp1* ortholog in *P. vivax* was amplified from 169 base pairs (bp) upstream of the N-terminal of PVX_096245 and analysed. A putative signal peptide encoded in the 5′ upstream section of the *pvralp1* gene sequence was found. Hence, to obtain the full-length *pvralp1* gene sequence, the full-length target gene from 169 bp upstream of PVX_096245 was amplified and sequenced, and the full-length PvRALP1 protein sequence comprising 675 amino acid (aa) residues was successfully annotated. In detail, the full-length PvRALP1 contains a signal peptide (aa 1–23), a Gly- and glutamate (Glu)-rich region (aa 301–387), and a Leu-rich region (aa 431–452) (Figure 1A). A Leu zipper domain [L_431_-(X)_6_-L-(X)_6_-L(X)_6_-L_452_] similar to that of PfRALP1, could not be predicted by the 2ZIP server [27] (Figure 1A). One coiled-coil α-helical motif (aa 517–546), which might be involved in protein–protein interactions, was identified (Figure 1A), and was characterized by heptamino acid repeats (abcdefg)*n* with hydrophobic residues located in positions a and d, and generally polar residues in the remaining sites.

### Expression of recombinant PvRALP1-Ecto and PvRALP1-Tr proteins using the WGCF system

To characterize the PvRALP1 protein and its particular structure, including the Gly/Glu-rich and Leu-rich domains, PvRALP1-Ecto (without signal peptide, aa 31–675) protein with a GST-tag and PvRALP1-Tr (aa 257–503) protein with a His-tag (Figure 1A) were expressed using the WGCF system. The recombinant PvRALP1-Ecto and PvRALP1-Tr proteins were expressed in the soluble fraction (Figure 1B and C; arrows) and purified by affinity chromatography. Although there was expression of other proteins in the crude lysate, causing the corresponding weak bands in the T, S, E1, and E2 lanes, the purified target proteins are clearly visible in lanes E3. These results demonstrated that the WGCF system is able to produce PvRALP1-Ecto and PvRALP1-Tr as soluble proteins.

### Recognition of recombinant PvRALP1 proteins

**Figure 2:**
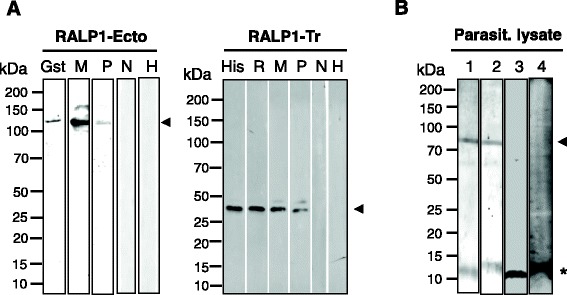
Western blot analysis of recombinant PvRALP1-Ecto, PvRALP1-Tr, and schizont lysate probed with antisera to PvRALP1-Ecto and PvRALP1-Tr, and sera from *P. vivax* malaria patients and healthy controls. **(A)** PvRALP1-Ecto and PvRALP1-Tr were probed respectively with anti-GST antibody (Gst), immune mouse sera (M), pooled *P. vivax* patient sera (P), anti-His antibody (His), immune rabbit sera (R), nonimmune mouse sera (N), and sera from noninfected humans (H). **(B)** Recognition of PvRALP1 antigen in the parasite lysate with mouse (1) and rabbit (2) antisera against PvRALP1-Tr, preimmune mouse sera (3), and preimmune rabbit serum (4), respectively. Arrowheads indicate the target bands for native and recombinant proteins and putative processed fragments. The asterisk indicates a nonspecific band present in each lane.

Recombinant PvRALP1-Ecto and PvRALP1-Tr proteins were used to immunize mice or a rabbit to produce polyclonal antibodies. Immune sera from these animals and those from *P. vivax*-infected humans specifically recognized PvRALP1-Ecto and PvRALP1-Tr (Figure 2A). However, neither preimmune animal sera nor sera from noninfected humans reacted with these antigens, suggesting that PvRALP1 induced specific responses in both animals and humans. To confirm whether these antisera recognized native PvRALP1 from the parasite, *P. vivax* schizont-enriched lysate was tested against mouse and rabbit anti-PvRALP1-Tr antibodies. Both anti-PvRALP1-Tr antisera (Figure 2B, lanes 1, 2; arrowheads) but not preimmune sera (Figure 2B, lanes 3, 4) recognized a band approximately 73 kDa in size. A nonspecific 12 kDa band (Figure 2B, lanes 3, 4, asterisk) from erythrocyte ghosts was detected by both antisera and preimmune sera.

### Analysis of humoral immune responses

**Figure 3:**
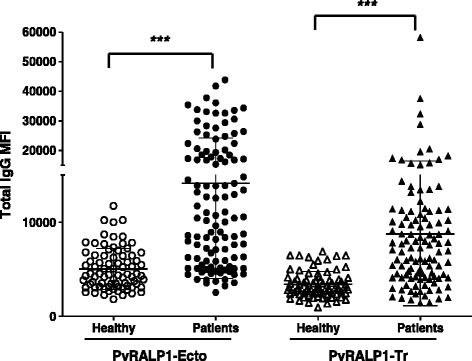
Total IgG antibody responses to PvRALP1-Ecto and PvRALP1-Tr assessed protein microarrays. Immunoreactivity of total IgG against each antigen was determined with the sera of malaria patients (Patients) and healthy individuals (Healthy) from the Republic of Korea. High specificity of responses and significant differences between *P. vivax* patients and healthy individuals were observed in the prevalence of total IgG specific for the two PvRALP1 antigens (*p* < 0.0001).

To further evaluate the humoral immune responses against PvRALP1-Ecto and PvRALP1-Tr, the antibody responses against these targets in serum samples from 112 patients infected with *P. vivax* and 80 healthy individuals were determined. The sera from *P. vivax*-exposed individuals showed significantly higher MFI than those from malaria-naïve subjects (Figure 3, *p* < 0.0001). The prevalence of anti-PvRALP1-Ecto and anti-PvRALP1-Tr antibodies showed that the sensitivities were 58.9% and 55.4% and the specificities were 95.0% and 92.5%, respectively (Table 1).

**Table 1:** **Prevalence (% positive), 95% confidence intervals, and mean fluorescence intensity of IgG responses to** ***Plasmodium vivax*** **RALP1-Ecto and RALP1-Tr in sera from human** ***P. vivax*** **patients and healthy individuals**

**RALP1**	**No. of patient samples (** ***n*** **)**			**95% CI** ^**b**^	**MFI (%)**	**No. of healthy individuals (** ***n*** **)**			**95% CI**	**MFI (%)**	***P*** **-value** ^**d**^
	**Positive**	**Negative**	**Total (%)** ^**a**^			**Positive**	**Negative**	**Total (%)** ^**c**^			
Ecto	66	46	112 (58.9)	49.67–67.6	14138	4	76	80 (5.0)	87.54–97.99	5032	*p* < 0.0001
Tr	62	50	112 (55.4)	46.13–64.23	8757	6	74	80 (7.5)	84.59–96.52	3047	*p* < 0.0001

MFI = mean fluorescence intensity.

^a^Sensitivity: % positive in patient samples.

^b^Confidence intervals.

^c^Specificity: % negative control healthy samples.

^d^Differences in the total IgG prevalence for each antigen between *P. vivax* patients and healthy individuals were calculated with Student’s *t*-test. *P* < 0.05 was considered significant.

**Figure 4:**
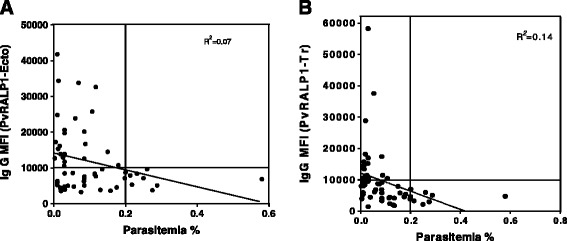
Correlation between peripheral blood parasitaemia in *P. vivax* patients and the immunoreactive fluorescence intensity of their sera. Correlation between immunoreactivity of total IgG against PvRALP1-Ecto **(A)** and PvRALP1-Tr **(B)** and the parasitaemia of each *P. vivax* patient sample was analysed for 56 *P. vivax* patient samples. *R* ^2^ was calculated by a polynomial program.

To clarify whether anti-PvRALP1 antibody levels correlate with effective immunity, the antibody responses against the two antigens were analysed in association with the individual levels of parasitaemia in 56 patients (Figure 4A and B). There was no significant inverse correlation between levels of parasitaemia and levels of antibody against either PvRALP1-Ecto (*R*^2^ = 0.11) or PvRALP1-Tr (*R*^2^ = 0.14) antigen. However, there were no patients with high antibody levels who also had high parasitaemia (Figure 4A and B, top right quadrant).

### Isotype distribution of the IgG response to PvRALP1 antigen in malaria patients and immunized mice

**Figure 5:**
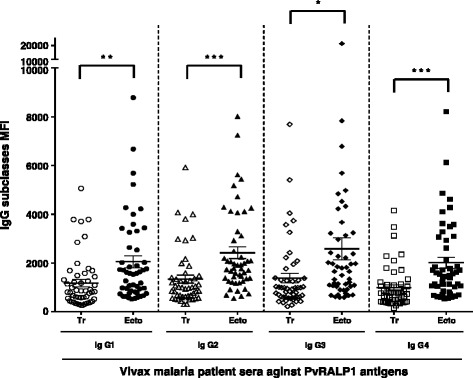
IgG subclass antibody responses to PvRALP1-Ecto and PvRALP1-Tr assessed protein microarrays. The *p* values were calculated using Student’s *t*-test. The bar indicates the mean ± standard deviation. MFI, mean fluorescence intensity. Single asterisk, 0.01 < *p* < 0.05; double asterisks, 0.0001 < *p* < 0.01; triple asterisks, *p* < 0.0001.

**Figure 6:**
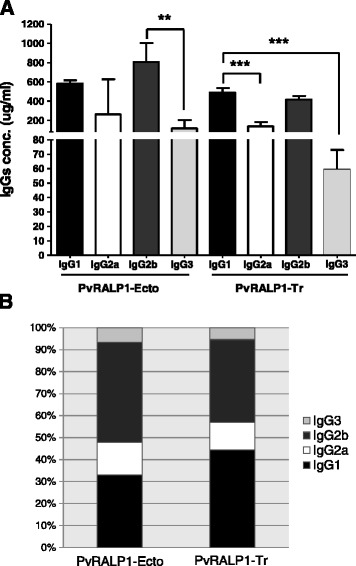
PvRALP1-specific IgG subclass levels and percentages in immune mouse sera. **(A)** IgG1 (black), IgG2a (white), IgG2b (dark gray), and IgG3 (light gray) levels after the final immunizations against PvRALP1-Ecto and PvRALP1-Tr were determined by ELISA. **(B)** Geometric mean concentrations were calculated for each of the four subclasses in each group of three mice and are presented as percentages of the total IgG responses in BALB/c mice. The results are expressed as mean titers ± SD. The *p* values, calculated using Student’s *t-*test, indicate the number of sera analysed. Double asterisks, 0.0001 < *p* < 0.01; triple asterisks, *p* < 0.0001.

Because protective antibodies against *Plasmodium* blood stages have been shown to belong to the cytophilic subclasses [28], the IgG subclass responses to PvRALP1-Ecto and RALP1-Tr antigens were analyzed. The mean levels of the IgG subclasses in the responses of humans to both antigens were ranked IgG3 > IgG2 > IgG1 > IgG4 (Figure 5), with no significant differences between the subclasses (*p* > 0.05). However, the levels of all IgG subclasses for PvRALP1-Ecto were significantly higher than those for PvRALP1-Tr (IgG1, *p* < 0.01; IgG2, *p* < 0.001; IgG3, *p* < 0.05; and IgG4, *p* < 0.001). A balanced Th1/Th2 response was observed in mice immunized with PvRALP1-Ecto and PvRALP1-Tr (IgG1/IgG2a ratios: 0.7 and 1.2). As shown in Figure 6A and B, IgG2b levels (mean concentration 805.0 μg/mL) were found to be highest in PvRALP1-Ecto-immunized mice and IgG1 levels (mean concentration 490.5 μg/mL) were highest in PvRALP1-Tr-immunized mice.

### Localization of PvRALP1 in the rhoptry neck of the merozoite

**Figure 7:**
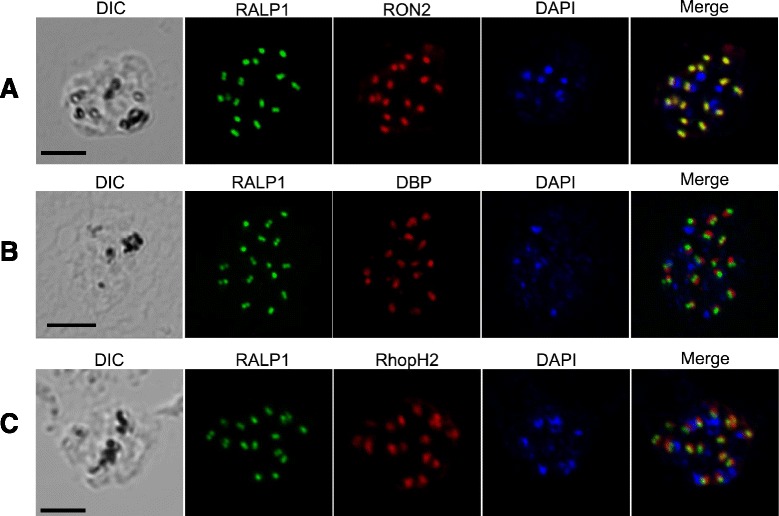
Subcellular localization of PvRALP1 protein in asexual blood-stage parasites of *P. vivax*. Acetone-fixed mature schizonts of *P. vivax* were dual labeled with mouse immune sera against PvRALP1-Tr (green), PvRON2 (rhoptry neck marker) (**A**, red), rabbit immune sera against PvDBP (microneme marker) (**B**, red), and PvRhopH2 (rhoptry marker) (**C**, red). Nuclei are visualized with DAPI (blue). DIC; differential interference contrast image. Scale bar represents 5 μm.

To determine the localization of the native PvRALP1 protein in merozoites of *P. vivax*, IFA was carried out on mature schizont stage *P. vivax* using anti-PvRALP1-Tr; anti-Duffy binding protein (DBP) as a microneme marker [29], anti-PvRhopH2 as a rhoptry body marker [8], and anti-PvRON2 as a rhoptry neck marker [30]. The IFA results showed that the apical distribution of PvRALP1, detected using anti-PvRALP1-Tr antiserum, clearly co-localized with that of PvRON2 (Figure 7A), but not with that of PvDBP (Figure 7B) or PvRhopH2 (Figure 7C). These results demonstrate for the first time that native PvRALP1 localizes in the rhoptry neck of merozoites, although immunoelectron microscopic observation is essential to confirm this.

## Discussion

In *P. falciparum*, anti-RALP1 antibodies inhibited the merozoite invasion of erythrocytes and disrupted MJ formation, which suggested that PfRALP1 is essential for erythrocyte invasion [13]. PfRALP1 localized in the rhoptry neck of the merozoite, and it translocated from the rhoptry neck to the MJ during merozoite invasion [13]. In *P. vivax* parasites, because of the difficulty of *in vitro* culture, the function of the analog is not easy to explore. However, the immune responses to and localization of PvRALP1 could be analysed in this study. The rhoptry neck localization of PvRALP1 in merozoites and its immune profiling in *P. vivax* malaria patients suggested that PvRALP1 may play a similar important role during the merozoite invasion process to that of PfRALP1 [14].

In a previous study, the Glu-rich region of some *Plasmodium* parasite proteins appeared to react strongly with human IgG antibodies [31,32]. The α-helical coiled-coil motifs were also targets of antibody recognition, suggesting that these antibodies may be protective against malaria vaccine candidates including liver stage antigen (LSA)-1 [33], LSA-3 [34], MSP-3 [35], MSP-6 [36], and MSP-1 [37]; such motifs have been used for rapid identification of malaria vaccine candidates [20]. Because of its structural similarity to PfRALP1, the immunogenicity of PvRALP1 was characterized by immune profiling of PvRALP1-Ecto and PvRALP1-Tr constructs including the Gly/Glu-rich and Leu-rich domains. Although there were no significant differences detected in either sensitivity or specificity of responses in sera from infected humans to each protein (Table 1), the intensity of IgG and IgG subclass responses to PvRALP1-Ecto were higher than those against PvRALP1-Tr, which indicates that motifs including a Gly/Glu-rich domain, a Leu-rich domain, and a coiled-coil region may be related to the immunogenicity of these proteins in infected patients.

A number of *pvralp1* genomic sequences from 170 isolates obtained worldwide are available in the PlasmoDB databases and analysis for alignments showed a comparatively conserved *pvralp1* gene. In addition, the total number of single-nucleotide polymorphisms (SNPs) in all *P. vivax* strains is 78, and the ratio of nonsynonymous/synonymous SNPs ratio is 2.12 (53/25). A small number of SNPs were detected that might be related to the low antibody response against the two recombinant PvRALP proteins that was observed in some patients’ sera.

Knowledge of the IgG subclass responses that are associated with protection against malaria is essential for understanding anti-malaria immunity and guiding vaccine research. IgG1 and IgG3 are the predominant subclasses produced in response to merozoite antigens in humans [38-41]. IgG1 and IgG3 are cytophilic and mediate phagocyte activation and complement fixation [42]. It has been suggested that IgG3 is more efficient at mediating these processes [42]. In the present study, both PvRALP1-Ecto and PvRALP1-Tr induced predominantly IgG3 antibodies (Figure 5), suggesting that PvRALP1 might induced functional antibodies. While the factors determining subclass responses to antigens are not clearly defined, antigen properties, host age, cumulative exposure, and genetic determinants have been linked with the nature of subclass responses [43-45].

The IgG subclass responses to these two antigens in immunized mice were evaluated, presuming that the noncytophilic IgG1 and IgG3 isotypes correspond to a Th2 response, whereas the cytophilic IgG2a and IgG2b correspond to a Th1 response [46]. The results showed that a balanced Th1/Th2 response was observed to both antigens, and that IgG1 and IgG2b responses were higher (Figure 6) in PvRALP1-immunized mice than any of the other IgG subclasses. A similar IgG subclass distribution has been found in responses in mice immunized with the vaccine candidate PfMSP3 [47], and these were able to inhibit parasite growth. An erythrocyte binding motif PvMSP1P-19 also induced predominantly IgG2b responses in immunized mice [3]. It has been reported that the function of mouse IgG2b is similar to that of IgG3 in humans [48], and immunoepidemiological studies have shown a significant correlation of human IgG3 antibody responses with protection acquired by natural exposure to the parasite [49]. There was no significant inverse correlation between the level of parasitaemia and antibody levels against either PvRALP1-Ecto or PvRALP1-Tr antigens, although there was also no patient with high antibody levels and high parasitaemia (Figure 4). These findings suggest that the level of anti-PvRALP1 antibodies does not strongly contribute to the degree of inhibition of parasite growth. Furthermore, the levels of IgG2b in PvRALP1-Ecto-immunized mice were paralleled the levels of human IgG3 in sera from *P. vivax*-infected patients (Figures 5 and 6).

In the present study, PvRALP1 localized to the rhoptry neck of merozoite parasites was observed (Figure 7): PvRALP1 completely colocalized with the rhoptry neck marker PvRON2. Hence, we have clearly demonstrated that PvRALP1 is likely to be a rhoptry neck protein of *P. vivax* merozoites, although an immunoelectron microscopy study should be performed to confirm this. During the invasion of the merozoite, rhoptry neck proteins (i.e., RON2, RON4, RON5, and RON8) are discharged from the rhoptries to form the MJ complex with a microneme protein, AMA1, and this complex plays an important role in the invasion of erythrocytes [50]. Thus, it is possible that, like other rhoptry neck proteins, PvRALP1 may also play an essential role in this invasion process.

## Conclusions

An investigation of immune responses to PvRALP1 and investigation of its localization suggested that PvRALP1 might play an important role in erythrocyte invasion by blood-stage *Plasmodium vivax* parasites. However, more functional and biological investigations of PvRALP1 are required.
